# Timing of Digital Device Exposure as a Source of Measurement Error in Dry Eye Disease Research: A Critical Narrative Review

**DOI:** 10.7759/cureus.114181

**Published:** 2026-08-08

**Authors:** Wasse Uddin Ahmed Saleh

**Affiliations:** 1 General Surgery, Nottingham University NHS Trust, Nottingham, GBR

**Keywords:** blink rate, computer vision syndrome, digital eye strain, dry eye disease, meibomian gland dysfunction, ocular surface, online learning, screen time, smartphone, tear film

## Abstract

Dry eye disease (DED) is a common, multifactorial disorder of the ocular surface, and rising digital device use is increasingly recognised as a modifiable contributor. Digital eye strain (DES), a related but distinct symptom construct that also encompasses uncorrected refractive error and binocular vision anomalies, is reported in 50-90% of regular device users, compared with 5-50% for DED itself depending on diagnostic criteria; these figures should not be conflated.

Reduced blink rate during screen viewing appears driven substantially by cognitive task demand rather than the screen itself, since a controlled comparison of matched digital and hard-copy reading found no difference in blink rate between formats. Incomplete blinking, however, was higher with the digital device and is independently associated with dry eye signs and symptoms in cross-sectional data, though that data did not measure device exposure and cannot establish that screen use causes the incomplete blinking seen in habitual users. No additional effect of contact lens wear on screen-related dryness was detected in one experimental study using 20-minute exposures; this is not evidence of no effect. Behavioural interventions, including the 20-20-20 rule, and oral omega-3 carry only low-certainty evidence, and a large randomised trial found omega-3 ineffective for dry eye more broadly.

The 2023 Tear Film and Ocular Surface Society (TFOS) Lifestyle report comprehensively reviewed this literature and identified an unanswered question: no study has systematically tracked how ocular surface measurements change over time after digital device exposure ends. This review takes that gap as its focus. It argues that recent, unstandardised device exposure before ocular surface testing is a source of measurement error that has not been controlled for across the dry eye literature, examines the evidence for this effect, and proposes a research design and, informed by imaging tools that have become available since 2022, a feasible way to close the gap.

## Introduction and background

Dry eye disease (DED) is defined by the Tear Film and Ocular Surface Society Dry Eye Workshop II (TFOS DEWS II) as a multifactorial disease of the ocular surface characterised by loss of tear film homeostasis, accompanied by ocular symptoms, in which tear film instability and hyperosmolarity, ocular surface inflammation and damage, and neurosensory abnormalities play aetiological roles [1]. TFOS DEWS III, published in 2025 across a digest and companion Diagnostic Methodology and Management and Therapy reports in the American Journal of Ophthalmology, reaffirms this framework and lists digital device use among the confirmed, modifiable environmental risk factors for DED, situating reduced blink function during screen use within the mechanism of evaporative dry eye [2-4].

The most comprehensive existing synthesis of this specific topic is the TFOS Lifestyle report on the impact of the digital environment on the ocular surface, a systematically searched and evidence-graded report covering screen types, blinking, dry eye signs, children, COVID-19, contact lenses, the 20-20-20 rule, blue light, and artificial tears [5]. 

The terms "digital eye strain" (DES) and "computer vision syndrome" (CVS) are frequently used interchangeably in the literature, but they are not synonymous. DES is the term now preferred by TFOS, because CVS implies a problem specific to computer displays, whereas the underlying visual and ocular symptoms occur across all digital device types [5]. It is also important to state plainly that a substantial proportion of DES arises from uncorrected refractive error and binocular vision anomalies rather than from the ocular surface at all [5]. DES and DED are therefore related but distinct constructs, and this review uses "digital eye strain" throughout, reserving "dry eye disease" for the ocular-surface-specific diagnosis.

Population-based estimates of DED prevalence vary enormously, from approximately 5% to over 50% of adults depending on whether diagnosis rests on symptoms alone, signs alone, or a combination of the two [6], and a 2021 Bayesian synthesis of studies published between 1997 and 2021 placed the global prevalence of symptomatic disease at approximately 9-12%, with marked variation by sex, age, and geographic region [7]. DES prevalence, by contrast, is reported at roughly 50-90% of regular users depending on population and threshold [8]; these two ranges describe different constructs and are kept separate throughout this review. Over the past two decades, the way people work, study, and socialise has been transformed by the proliferation of smartphones, laptops, tablets, and other digital displays, and this shift in visual behaviour has emerged as a distinct and highly prevalent risk factor for DED that is largely absent from the classical epidemiological literature predating the smartphone era.

Early work on visual display terminal (VDT) use and the ocular surface, conducted principally in Japanese office workers, demonstrated that individuals who used computer screens for extended periods at work had a substantially higher prevalence of clinically diagnosed dry eye than the general population, with prevalence estimates of definite or probable DED reaching approximately 10% and symptomatic complaints far higher [9,10]. Subsequent systematic reviews and meta-analyses have reinforced that VDT use is a consistent risk factor for DED across diverse occupational and cultural settings, although with considerable between-study heterogeneity [11]. This review’s English-language search also under-represents this Japanese-language foundational literature, a limitation returned to below.

Review methods and scope

This is a critical narrative review rather than a formal systematic review, though its literature base was assembled deliberately. PubMed/MEDLINE and Google Scholar were searched using combinations of the terms "dry eye," "digital eye strain," "computer vision syndrome," "visual display terminal," "smartphone," "tablet," "e-reader" / "electronic paper," "blink rate," "meibomian gland dysfunction," "contact lens," "children," and "COVID-19," restricted to English-language publications. Screening and selection were carried out by a single author. Embase, the Cochrane Library, Scopus, and Web of Science were not searched, and the English-language restriction excludes a substantial body of foundational Japanese-language work in this field, including antecedents of Uchino et al., 2008, and Uchino et al., 2013 [9,10]; both are acknowledged as limitations. Priority was given to systematic reviews and meta-analyses where available, supplemented by primary experimental and observational studies illustrating mechanism, device-specific effects, paediatric populations, and management. 

What This Review Adds

The TFOS Lifestyle report provides a comprehensive, systematically searched, evidence-graded synthesis of digital device use and the ocular surface, and this review does not seek to duplicate it [5]. That report’s search closed in November 2022 and identified, as an explicit open question, that no study had systematically tracked the time course of symptom onset after digital device exposure, or how this varies between individuals [5]. The present review takes that gap as its focus: it examines evidence that recent digital device exposure produces short-term, transient changes in ocular surface measurements, considers the implications for the timing of clinical assessment and for dry eye research design, and reviews literature published since the TFOS search date, including new objective measurement tools that make closing this gap feasible. A related 2025 review addressed the need for objective assessment in digital eye strain but focused on measuring the exposure, that is, quantifying screen time itself [12]. The present review instead concerns measuring the outcome: the finding that the ocular surface test result itself is time-dependent relative to recent screen use. These are distinct problems, and both are worth solving.

## Review

Epidemiology: global, regional, and occupational variation

Reliable quantification of how common digital-device-associated dry eye actually is remains surprisingly difficult, and the literature illustrates why. The TFOS DEWS II Epidemiology Report catalogued prevalence estimates spanning roughly 5% to 50% across studies using different diagnostic criteria, with sign-based diagnoses generally yielding higher and more variable estimates than symptom-based ones [6]. Applying a Bayesian meta-analytic approach to 30 studies published between 1997 and 2021, Papas estimated a global prevalence of symptomatic DED of approximately 9-12%, with prevalence roughly 40% higher in women than men and striking geographic variation, from under 5% in some North American cohorts to over 30% in parts of Asia [7]. A 2022 systematic review and meta-analysis focused specifically on the United States reached a more cautious conclusion: rather than offering a single confident prevalence figure, the authors explicitly stated that prevalence and incidence estimates for both DED and meibomian gland dysfunction (MGD) remain difficult to pin down because of inconsistent case definitions and a lack of standardised diagnostic criteria across the primary literature, a methodological critique that applies with equal force to the digital-device-specific literature reviewed here [13]. A parallel meta-analysis of global MGD prevalence found comparably wide variation, from under 20% to over 60% depending on population and diagnostic approach, reflecting the same underlying problem of definitional inconsistency rather than necessarily true differences in disease burden [14]. TFOS DEWS III has since taken a concrete step toward resolving this specific problem, recommending a single short questionnaire, Ocular Surface Disease Index-6 (OSDI-6), in place of the mixture of instruments used previously [3]. This is a genuine, partial standardisation of the field and is discussed further in the Diagnostic Considerations section below.

Against this backdrop of substantial baseline heterogeneity in how DED itself is measured, studies of digital device use have generally identified screen exposure as an additional driver of prevalence, though the size of the reported effect varies with occupational context, geography, and study design, and this attribution requires an important caveat. The first large-scale evidence came from Japan, where cross-sectional surveys of office workers using VDTs found clinically diagnosed DED in approximately one in 10 workers, with longer VDT working hours associated with greater likelihood of disease, and tear film instability as the dominant clinical sign [9]. A related Osaka cohort similarly identified extended VDT hours as an independent risk factor [10]. However, a large population-based study described in the TFOS Lifestyle report found that the elevated dry eye risk observed in office-based occupations was no longer apparent after adjustment for 45 dry-eye-related conditions, with contact lens wear the single most important confounder; after adjustment, building and machinery workers, not office workers, carried the highest risk [5]. This finding substantially qualifies any description of screen exposure as an independent driver of occupational dry eye prevalence, and that phrase is used advisedly, and only where adjusted analyses support it, throughout this review.

A multinational systematic review and meta-analysis of VDT-related dry eye confirmed that VDT users have a significantly higher prevalence of DED than non-users, but the authors noted substantial statistical heterogeneity across the pooled studies, likely reflecting differences in occupational exposure definitions, diagnostic thresholds, and unmeasured confounders such as ambient humidity and air conditioning [11]. Two companion reviews published in Acta Ophthalmologica in 2022 attempted a more systematic synthesis of this literature: one focused on the epidemiological association between VDT use and dry eye [15], and a companion paper focused specifically on the pathophysiological mechanisms proposed to underlie it [16]; both concluded that while the direction of association is consistent, the primary studies they reviewed were overwhelmingly cross-sectional and relied on self-reported exposure, limiting causal inference. A further narrative review proposed a "vicious cycle" model in which VDT-induced blink changes lead to hyperosmolarity, inflammatory mediator release, and goblet cell loss, which in turn further destabilises the tear film [17]; this concept originates in the broader dry eye literature, where it is generally credited to Baudouin and colleagues as the vicious circle of dry eye, rather than being specific to display use, and the review acknowledged that the model is derived largely from cross-sectional associations rather than longitudinal confirmation.

Occupational and student surveys report an even wider range of DES prevalence, from approximately 50% to over 90% [8,18,19], a spread that is difficult to interpret without accounting for the instrument used to define a case. The validated computer vision syndrome questionnaire (CVS-Q), for instance, applies a specific symptom-frequency-and-intensity scoring threshold that differs substantially from the simpler yes/no symptom checklists used in other surveys, and studies using CVS-Q have found a clear dose-response relationship between hours of screen exposure and symptom severity that is less consistently reported using cruder instruments [19]. Part of this spread also reflects genuine construct overlap: many CVS-Q symptoms are shared with ocular allergy, infection, contact lens wear, and systemic conditions, any of which will independently raise the score [5]. Taken together, the epidemiological literature supports a real and biologically plausible association between digital device use and DED, but the precise magnitude of that association cannot currently be stated with confidence, and readers should treat any single prevalence figure quoted in this literature with appropriate caution.

Pathophysiology: blink dynamics, tear film instability, and meibomian gland dysfunction

The mechanistic link between digital device use and DED centres on changes in blinking behaviour during visually demanding, cognitively absorbing near tasks. Under normal, task-dependent conditions the spontaneous blink rate is approximately 15-20 blinks per minute, but multiple studies have documented a marked reduction - often by half to two-thirds - during sustained screen viewing, alongside a rise in the proportion of incomplete blinks, in which the upper eyelid fails to make full contact with the lower lid margin [8,15]. This reduced and incomplete blinking shortens the interval over which the tear film is replenished and re-spread across the ocular surface, allowing localised areas of the cornea and conjunctiva to remain exposed for longer, which accelerates evaporation of the aqueous tear component and destabilises the pre-corneal tear film [8].

This mechanistic picture requires an important qualification. A controlled comparison, reported in the TFOS Lifestyle report, of 20 minutes of reading from a screen and from hard copy, matched for text size, contrast, luminance, and viewing angle, found no difference in blink rate between the two formats; incomplete blinking, however, remained higher with the digital device [5]. This finding suggests that the fall in blink rate documented across the wider literature is driven substantially by the cognitive demand of the reading task rather than by the screen itself, whereas blink completeness may be a more display-specific effect. Blink rate and blink completeness are therefore treated as separable outcomes with potentially different drivers throughout the remainder of this review.

The strongest direct clinical evidence linking incomplete blinking to measurable disease comes from an age-, sex-, and ethnicity-matched cross-sectional study of 154 adults, in which participants with clinically detectable incomplete blinking were more than twice as likely to meet TFOS DEWS II dry eye diagnostic criteria (64% versus 44%; odds ratio 2.2, 95% confidence interval 1.2-4.2) and had significantly higher OSDI scores and greater meibomian gland dropout on meibography than those with complete blinking [20]. This finding is important because it moves beyond the purely correlational, screen-exposure-based literature to demonstrate a direct, measurable relationship between blink completeness itself and both symptomatic and structural markers of disease, independent of any particular device. It also, however, illustrates a genuine limitation in the broader mechanistic narrative: because the study did not itself measure digital device exposure, it demonstrates that incomplete blinking is associated with dry eye, without directly proving that screen use is the cause of the incomplete blinking observed in habitual device users, as opposed to some other underlying trait or behaviour. This caveat is carried through to the abstract and conclusion of this review.

A direct experimental demonstration of the screen-specific pathway comes from studies of high-intensity smartphone reading, in which two hours of continuous smartphone use was sufficient to produce a statistically significant fall in tear breakup time, an increase in ocular surface redness, and a corresponding rise in both the diagnostic rate and severity of dry eye on standardised clinical testing, illustrating how quickly screen-induced blink changes can translate into measurable ocular surface compromise [21]. Comparable acute effects have been observed with smartphone reading under different display technologies and lighting conditions, with organic light-emitting diode (OLED) screens producing greater reductions in tear stability and greater increases in symptom scores than electronic-ink displays, suggesting that display type and luminance, in addition to viewing duration, modulate the magnitude of ocular surface stress [22,23]. Similarly, changes in tear film lipid layer quality, tear meniscus height, and ocular surface symptoms have been documented within as little as 20-60 minutes of smartphone reading or video-game play in healthy young adults, indicating that ocular surface disturbance from digital devices can occur acutely, well before any chronic structural change develops [24,25]. The largest recent paediatric dataset, a study of 462 school children using tear breakup time and Schirmer testing, found a dose-response effect above approximately 3-3.5 hours of device use per day and an odds ratio of 1.94 for each additional half-hour of computer use; critically, in the same children, time spent reading physical books was associated with lower OSDI scores and better ocular surface health [26]. This is currently the strongest available evidence that the display itself, rather than close reading alone, is doing something, and it is discussed further in the paediatric section below.

Beyond these acute, largely aqueous-evaporative effects, there is growing evidence that chronic alterations in blink completeness contribute to secondary MGD, a leading cause of evaporative dry eye. Because complete blinking is required to mechanically express meibum from the meibomian glands and distribute it across the tear film lipid layer, persistent incomplete blinking is thought to reduce lipid secretion and promote gland stasis and, over time, gland dropout [8,20]. Clinical studies in populations with impaired or incomplete blinking, such as patients recovering from facial nerve palsy, have directly demonstrated that a sustained pattern of incomplete blink closure is associated with measurable MGD, providing mechanistic support, drawn from a non-screen-related population, for the hypothesis that habitual incomplete blinking during digital device use could similarly predispose regular users to lipid-deficient, evaporative dry eye over time [27]. This comparison should be read as illustrative rather than equivalent: facial nerve palsy involves lagophthalmos and orbicularis oculi denervation, a structural, paralytic failure of eyelid closure, which differs fundamentally from the voluntary, cognitively driven suppression of blink completeness seen during screen use, and the facial-palsy literature can only be taken as evidence that incomplete closure of any cause impairs meibomian gland function, not as a direct model of the VDT-associated mechanism itself. Consistent with this, occupational studies of VDT workers have found that the severity of dry eye symptoms and signs in this population correlates with the degree of underlying MGD, rather than with aqueous deficiency alone [28]. The TFOS Lifestyle report adds a directly relevant occupational finding: long-term computer workers, averaging approximately eight hours a day over eight years, showed more severe MGD and gland loss than shorter-term users [5]. However, a genuine limitation running through this entire mechanistic strand is that the facial-palsy evidence, the general-population blinking study, and the VDT-worker studies each examine a different population and a different exposure, and no single study has yet followed habitual, healthy digital device users longitudinally from normal meibomian gland structure through to demonstrable gland dropout, so the causal chain - screen use, to reduced/incomplete blinking, to chronic MGD - remains inferred from separate pieces of evidence rather than established directly [16,20,27,28].

Device-specific and comparative evidence: computers, smartphones, tablets, and e-readers

While the mechanistic pathway of reduced and incomplete blinking is shared across digital devices, the magnitude of ocular surface effect appears to vary with device type, largely as a function of viewing distance, gaze angle, and display technology, though direct head-to-head evidence remains sparser than the volume of single-device studies might suggest. Reading from a smartphone typically involves a closer working distance and a more downward gaze than reading from a desktop monitor. A lower gaze angle narrows the palpebral aperture and reduces exposed ocular surface area; the TFOS Lifestyle report quantifies this at approximately 1.2 cm² exposed when reading a book in downgaze, roughly double that for computer use, and almost triple in upgaze [5]. Handheld devices may therefore be less damaging than desktop monitors by this specific mechanism, even though the closer working distance associated with handheld use works in the opposite direction; TFOS states that, in general, computer use produces more signs and symptoms than smartphone use [5]. Comparative studies have found that ocular surface parameters, including tear osmolarity and conjunctival redness, differ measurably between computer- and smartphone-based reading tasks, with some studies reporting greater disturbance with computer use and others with handheld devices, consistent with these competing factors being task- and device-specific rather than uniform [24,29]. A narrative review focused on the evidence for ocular and visual discomfort across smartphones, tablets, and computers concluded that although symptomatic complaints are common with all device types, direct comparative data remain limited and the relative contribution of each device to cumulative ocular surface stress in individuals who use multiple devices throughout the day is not yet well characterised [30].

The electronic-paper literature, which predates much of the smartphone-specific research and which the TFOS Lifestyle report covers in detail, provides useful comparative evidence. In a longitudinal, within-subject study comparing a liquid crystal display (LCD) tablet, an electronic-paper (e-paper) reader, and a paper book across repeated prolonged reading sessions, reading on the LCD device produced significantly higher subjective visual fatigue and a greater reduction in spontaneous eye blink rate than either the e-paper device or paper, while e-paper was statistically indistinguishable from paper on these measures [31], consistent with TFOS’s own conclusion that reading behaviour is generally comparable between electronic paper and printed paper [5]. This finding is important for critical interpretation of the wider literature because it suggests that at least part of the ocular surface burden attributed to "digital devices" in general may be attributable specifically to backlit, higher-luminance LCD and OLED technology rather than to screen-based reading per se, a distinction that most smartphone- and computer-focused studies, which rarely include a paper or e-paper comparison arm, are not designed to test [22,23,31]. Consistent with this, comparative studies of different smartphone display technologies have similarly found that OLED screens are associated with greater tear film disturbance than e-paper alternatives under otherwise matched reading conditions [22]. This should not, however, be read as attributing the effect to display technology in isolation: TFOS cites a study finding that blue light emission was essentially independent of display technology and instead closely tracked the display’s correlated colour temperature [5], a finding this review’s underlying argument, that backlit high-luminance displays carry a distinct burden, must be read alongside rather than in place of.

Experimental studies isolating smartphone use have found that even a single session of gaming or reading on a smartphone screen produces immediate reductions in blink rate and tear film stability alongside worsened subjective symptoms in adults without pre-existing dry eye, supporting a direct, dose-dependent pathway rather than mere association [24,25]. A systematic review with narrative synthesis of the smartphone-DED literature identified a broadly consistent positive association between smartphone use and DED across cross-sectional, case-control, and experimental study designs, while explicitly noting substantial heterogeneity in exposure measurement - self-reported hours versus objectively logged use - and in outcome definitions across the included studies, and recommending that future research adopt objective, device-based exposure logging rather than relying on recall [29]. A separate narrative review reached a similar conclusion regarding the relationship between DED and digital screen use more broadly, characterising the evidence as consistent in direction but heterogeneous in magnitude and quality [32].

Virtual and augmented reality headsets, though largely absent from the earlier digital-device literature, are covered by the TFOS Lifestyle report and merit brief mention. Interestingly, TFOS reports that headset wear improved lipid layer grade and tear film stability compared with a conventional display, plausibly because the enclosed, warmer microenvironment promotes meibum secretion [5], a useful counterexample to a simple "screens are harmful" narrative. A 2025 study using an ultra-compact camera for time-course, noninvasive observation of tear film dynamics during 30 minutes of VR gameplay found a significant increase in tear film lipid layer interference grade and periocular surface temperature as gameplay progressed, consistent with thickening of the lipid layer and, tentatively, improved rather than worsened tear film stability under these conditions [33]; this study is itself an example of the kind of time-course measurement this review argues is needed more broadly, and is discussed further below.

Contact lens wear and concurrent digital device use

Because contact lens wear and digital device use are each independently associated with ocular surface symptoms, and because many patients are exposed to both simultaneously, an important applied question is whether the two exposures combine additively. A randomised, within-subject experimental study assessed dry eye symptoms, tear meniscus height, non-invasive tear breakup time, conjunctival redness, and pupil size before and after 20-minute reading tasks performed on a computer and a smartphone, both with and without disposable contact lens wear, and with and without artificial tear instillation [34]. Reading on either device without lenses produced significant post-task increases in symptoms and conjunctival redness and, for the computer task specifically, a significant reduction in tear breakup time. Contrary to what might be expected, disposable contact lens wear did not produce a statistically significant additive effect on dry eye signs or symptoms beyond that of digital display use alone during these short exposures, while instillation of artificial tears was effective at mitigating the impact of display use regardless of lens wear status [34].

This is consistent with, rather than a new finding relative to, the TFOS Lifestyle report’s own conclusion that modern soft contact lens wear has not been found to increase DES symptom frequency or severity [5]. It warrants critical qualification rather than uncritical acceptance. The study examined only a single 20-minute exposure in young, healthy volunteers wearing daily disposable lenses, and a study that fails to detect an effect is not equivalent to a study demonstrating there is none, unless it was designed and powered for equivalence. It remains uncertain whether the same absence of an additive effect would hold for habitual, multi-hour daily device users, for older contact lens materials or reuse schedules more prone to deposit accumulation, or over the cumulative course of a full working day combining both exposures repeatedly. The authors themselves highlighted that screen position may be particularly relevant for contact lens wearers, since hand-held devices held closer and at lower gaze angles may cause less tear film disruption than desktop monitors viewed at a more open palpebral aperture, a nuance that has not yet been tested directly in contact lens wearers using naturalistic, full-day exposure protocols [34]. No additional effect was detected in this single experimental study using short exposures; that is the claim this review makes, in the abstract and elsewhere, rather than a claim that no effect exists.

Special populations: children, adolescents, and the COVID-19 online-learning experience

Children and adolescents represent a population of particular concern because screen exposure now begins earlier in life, may be sustained over decades, and children may be less able than adults to recognise and report early ocular discomfort. Early paediatric studies found that duration of video display terminal use was independently associated with DED in school-aged children, with tear breakup time inversely related to hours of daily device exposure [35]. A larger case-control study of over 900 children similarly identified smartphone use as an independent risk factor for paediatric DED, with the association varying by region and age, and with urban children - who tended to report higher rates of smartphone use - showing a higher prevalence of DED than their rural counterparts [36]. Experimental work in school-aged children has shown that even a single hour of smartphone gaming produces an immediate and sustained reduction in blink rate to roughly one-third of baseline values, accompanied by worsened ocular comfort, findings that closely parallel those previously reported in adults and suggest that the acute mechanistic pathway linking screen use to blink disruption operates similarly across the lifespan [37]. A recent narrative review of DED in the young similarly identified digital device use as one of the principal modifiable contributors to the increasing recognition of DED in paediatric and adolescent populations, alongside reduced outdoor time and increased near-work more broadly [38]. As detailed above, the largest and most recent paediatric dataset, a study of 462 school children using tear breakup time and Schirmer testing, found a dose-response threshold above approximately 3-3.5 hours of daily device use and, importantly, a protective association between time spent reading physical books and ocular surface health in the same children [26]; this is the most direct paediatric evidence that the display itself, rather than near work generally, contributes to disease.

The COVID-19 pandemic produced an unplanned, large-scale natural experiment in screen exposure among children, as school closures shifted instruction to online platforms accessed predominantly via smartphones, tablets, and laptops. A dedicated series of studies examining DES among children during online e-learning (the DESK studies) found a high prevalence of symptoms directly related to the number of hours spent on digital devices, with symptom scores rising in proportion to cumulative screen time [39], and a related follow-up study from the same research group found that the intensified digital device exposure associated with online classes and home confinement was also linked to accelerated myopia progression in children, underscoring that screen-related visual morbidity in this population extends beyond the ocular surface alone [40]. Both DESK studies were questionnaire-based, conducted at a single centre, and did not include clinical examination, which should temper the weight given to them; the myopia finding by Mohan et al., 2022, is also a departure from this review’s ocular-surface scope, retained because it illustrates the breadth of screen-related visual morbidity in children and because it is now supported by a 2025 dose-response meta-analysis linking digital screen time to myopia [40,41]. Collectively, this body of pandemic-era evidence reinforced pre-pandemic findings regarding digital device use and paediatric DED while highlighting how rapidly ocular surface symptoms can emerge when screen exposure increases sharply within a population that had not previously been accustomed to such intensive device use [37,39,40]. A limitation shared by this entire pandemic-era literature, however, is the difficulty of disentangling the effect of increased screen time itself from co-occurring changes during lockdown periods - including reduced outdoor time and natural light exposure, altered sleep patterns, indoor air conditioning, and psychological stress - any of which could independently affect the ocular surface and confound the apparent screen-time association.

Diagnostic considerations: the timing of exposure as a source of measurement error

This section contains the central argument of this review.

A practical implication of the mechanistic and experimental literature above is that recent, high-intensity digital device use can transiently inflate measures of dry eye severity independent of a patient’s baseline ocular surface status. Experimental work in which healthy volunteers underwent standardised ophthalmic examination before and after a period of high-intensity smartphone reading found that the diagnostic rate of dry eye rose sharply after just two hours of use, and that a proportion of participants who did not meet dry eye criteria at baseline did so afterward, indicating that recent device use can produce a false elevation in measured disease severity if testing is not appropriately timed [21]. This has direct implications for both clinical assessment and research design: if the interval between digital device exposure and ocular surface testing is not standardised, a study may be measuring a transient, device-induced tear film disturbance rather than a patient’s underlying chronic disease state [20,21].

This is compounded by a further, independent problem: the correlation between dry eye signs and symptoms is poor. In a cross-sectional study of 189 civil aircrew personnel, among the 87.3% who reported no subjective dry eye symptoms on a standard symptom questionnaire, 18.8% had an abnormal Schirmer test and 17.6% an abnormal tear breakup time [42]. A measurement taken at the wrong time, in a discipline where signs and symptoms already diverge substantially, compounds rather than merely adds to the field’s existing measurement uncertainty.

The TFOS Lifestyle report, the most comprehensive existing synthesis of this literature, states explicitly, in its section on blinking abnormalities, that no study has systematically tracked the time course of when symptoms first occur after digital device exposure, or how this varies between individuals [5]. This review treats that statement as its central justification: the leading report in the field identifies this specific measurement question as open, and this review argues that it can now be closed.

It could not have been closed in 2022. The objective measurement tools required did not yet exist at a scale suitable for repeated, standardised testing. They exist now. A deep-learning-assisted blinking analysis system has been validated against the LipiView interferometer in dry eye patients, showing better measurement consistency than the existing device on repeated testing [43]. A smartphone-based deep learning algorithm for tear meniscus height measurement, trained on over 1,000 images and achieving over 95% accuracy, has since been described [44]. A 2025 review surveys the broader state of deep-learning approaches to blink detection [45]. And blink rate has already been recorded in 456 people using an ordinary tablet camera with automated software, demonstrating feasibility of this kind of measurement at scale [46]. A related, independently reported finding lends further support to the timing argument: intraocular pressure has been shown to increase significantly at specific intervals after screen use [47], indicating that screen-related physiological change is not confined to the tear film and that interval-dependent effects are a general feature of this exposure. The 2025 study of tear film dynamics during VR headset use, noted above, is itself an example of exactly this kind of time-course measurement, applied to a different device category [33].

The study that should now be done is a repeated-measures design: standardised digital device exposure, followed by serial ocular surface measurements, using these newer objective tools, at fixed intervals after exposure ends, characterising both the magnitude and the duration of the shift, ideally across device types and in both adults and children. This would establish an evidence-based minimum pre-test abstinence interval for dry eye research and clinical assessment, analogous to fasting intervals in metabolic testing.

Two further points bear on how this timing problem should be addressed by clinicians and researchers today, pending that study. First, TFOS DEWS III has revised the diagnostic approach and now recommends OSDI-6 as a standardised instrument; recommendations about history-taking and assessment in this review are aligned to that current standard rather than to the 2017 DEWS II approach [3]. Second, given the high background prevalence of digital device use in virtually all age groups, a thorough dry eye history should routinely include quantification of daily screen time across all device types, typical viewing distance, and the presence of symptom exacerbation during or immediately after device use, since these factors may guide both diagnostic interpretation and the timing of subsequent testing [8,30].

Prevention and management strategies: a critical appraisal

Management of digital-device-associated dry eye follows the general principles applied to DED more broadly, emphasising behavioural modification, environmental optimisation, and tear film supplementation, with a growing evidence base evaluating the comparative efficacy of specific interventions, albeit one that remains, on close inspection, less robust than is sometimes assumed in patient-facing materials [48]. The most widely recommended behavioural strategy is the "20-20-20 rule," which advises users to pause every 20 minutes to view an object approximately 20 feet away for at least 20 seconds, with the goal of interrupting sustained near fixation and prompting a return toward normal blink frequency [8]. This recommendation currently rests on weaker evidence than is generally assumed. Talens-Estarelles et al., 2022, often cited in support of it, tested artificial tears, conscious blinking, and a blue-light overlay, but did not test scheduled breaks [51]. Two studies test the rule directly. One found that 20-second breaks every 20 minutes were not effective [49]. The other, from a group cited elsewhere in this review, found that symptoms improved with reminders but returned to baseline approximately one week after the reminders stopped [50]. The TFOS Lifestyle report further notes that an earlier study reporting benefit from scheduled breaks did not monitor compliance, so the apparent effect could reflect a placebo response [5]. The 20-20-20 rule should therefore be presented to patients as a plausible, low-risk habit rather than as an evidence-supported intervention, and this qualification applies wherever the rule is mentioned in this review, including in the abstract and conclusion.

Controlled studies isolating individual short-term interventions during simulated digital display tasks have found that both the instillation of artificial tears and conscious blink control produced measurably better ocular surface outcomes than a no-intervention control condition, whereas the use of a blue-light-filtering screen overlay conferred no additional benefit over control and, in some measures, performed worse than artificial tears or blink control [51]. In this study, blink control was one of five randomised, within-subject conditions (alongside a no-intervention control, artificial tears, a scheduled break, and a blue-light filter) assessed before and after a 20-minute laptop reading task in 47 healthy participants; the published abstract does not specify the exact instructional or reminder mechanism used to prompt conscious blinking during the task, and this operational detail should be confirmed against the original paper’s full methods section before being cited as a specific protocol.

A comprehensive systematic review and meta-analysis of randomised controlled trials evaluating interventions for CVS provides the most rigorous appraisal of this evidence base to date, and its conclusions are notably more circumspect than much of the preceding narrative literature: using the Grading of Recommendations, Assessment, Development, and Evaluation (GRADE) framework, the authors found that no single therapy was supported by high-certainty evidence, that low-certainty evidence favoured oral omega-3 fatty acid supplementation for reducing dry eye symptoms in symptomatic computer users, and that evidence for blue-light-blocking spectacle lenses was weak or absent [52]. This is the only intervention in this review’s strongest management source with any positive certainty rating, and it is reported here, in the abstract, and in the conclusion for that reason. It should be read alongside the DREAM trial, the largest randomised trial of omega-3 supplementation in DED generally, which was negative for its primary outcome [53]; the two findings pull in different directions. A subsequent Cochrane review of 17 randomised trials similarly found that blue-light-blocking lenses probably make no difference to eye strain [54]. The TFOS Lifestyle report additionally identifies secretagogues, warm compresses, humidity goggles, and ambient humidifiers as promising but less extensively studied options [5].

The low certainty ratings assigned throughout this evidence base reflect recurring problems across the underlying trials, including small sample sizes, short follow-up periods, difficulty blinding participants to behavioural interventions, and inconsistent outcome measures between trials, limitations that mirror the epidemiological heterogeneity discussed earlier in this review and that should temper confidence in any specific management recommendation [52]. These findings collectively suggest that simple, low-cost behavioural measures - deliberate, complete blinking; regular screen breaks; optimised viewing distance and downward gaze angle to reduce ocular surface exposure; adequate ambient humidity; and appropriate use of lubricating eye drops - currently rest on a firmer, if still modest, evidence base than optical filtering technologies marketed specifically for DES [48,51,52].

One further point does not appear elsewhere in this review and is arguably the single most useful practical recommendation in this field for a general medical readership: DES symptoms are considerably worsened by uncorrected or under-corrected refractive error, and as little as 0.50-1.00 dioptre of uncorrected astigmatism can significantly increase symptoms [5]. Refractive correction is commonly the appropriate first-line management step, ahead of behavioural or environmental measures, and should be considered before other interventions in any patient presenting with DES.

The TFOS DEWS III Management and Therapy report, published in 2025, has superseded the 2017 DEWS II framework cited previously in this literature and should now guide staged management: it emphasises subtyping of DED and recommends reducing controllable risk factors, explicitly including screen-related reduced blink rate and completeness [4]. Applied to the digital-device context, this suggests that patients with mild, exposure-related symptoms may benefit substantially from education about blinking, screen ergonomics, and refractive correction alone, whereas those with more persistent symptoms, or with objective signs of MGD, are likely to require a more comprehensive management approach directed at the underlying evaporative component of their disease, including lid hygiene and, where appropriate, targeted anti-inflammatory or meibomian gland-directed therapies, rather than digital device modification in isolation [4,27,28]. For contact lens wearers specifically, the available experimental evidence suggests that artificial tear instillation remains an effective, low-risk first-line strategy regardless of whether symptoms arise from lens wear, screen use, or their combination, pending further research into longer-term combined exposure [34].

For children specifically, the evidence reviewed above supports extending these same principles into paediatric practice and into educational settings that increasingly rely on digital instruction, including structured screen breaks during lessons, attention to screen distance and downward gaze angle, and parental or institutional monitoring of cumulative daily device exposure across both academic and recreational use [37,39,40]. Because children may not spontaneously report ocular discomfort, proactive screening for dry eye symptoms in paediatric patients with high reported screen time, rather than reliance on unprompted symptom report alone, is likely to improve early identification of at-risk children.

Critical appraisal of the evidence base and directions for future research

Drawing the preceding sections together, several cross-cutting methodological weaknesses recur throughout the digital-device-and-dry-eye literature and should temper how confidently its overall conclusions are held:

The overwhelming majority of epidemiological studies linking digital device use to DED are cross-sectional, which precludes firm conclusions about the direction of causality and leaves open the possibility that individuals with pre-existing ocular surface symptoms alter their device use patterns, rather than device use alone driving disease onset [11,15,29].

Exposure measurement is frequently based on self-reported screen time, which is subject to recall bias. This is not merely an assumption: a pre-registered meta-analysis of 106 effect sizes found that self-reported media use correlates only moderately with device-logged measurements [55]. Self-report also does not capture variation in viewing distance, gaze angle, blink behaviour, or device type that the mechanistic literature suggests are all clinically relevant modifiers of risk; several of the systematic reviews discussed above explicitly called for objective, device-logged exposure data in future work [29,30].

Case and outcome definitions vary widely between studies, spanning purely symptom-based questionnaires (which themselves differ, from the CVS-Q to the OSDI to the 5-item Dry Eye Questionnaire), sign-based clinical testing, and combined criteria; a 2022 meta-analysis focused on US prevalence data explicitly identified this definitional inconsistency as the central barrier to obtaining reliable prevalence and incidence estimates [13], a problem that propagates directly into the digital-device-specific literature reviewed here. The field has since moved, in part: TFOS DEWS III now recommends OSDI-6 as a single standard questionnaire, which should reduce, though not eliminate, this source of heterogeneity in future work [3].

Relatively few studies have followed device users longitudinally over months to years to determine whether the acute, experimentally demonstrable changes in blink rate and tear film stability translate into permanent structural changes such as meibomian gland dropout, or whether these effects are fully reversible with reduced exposure or behavioural intervention [16,20,28]. This imbalance is stark: at least 10 of the experimental studies discussed in this review assessed only a single, short-duration exposure ranging from 20 minutes to two hours [5,12,21-25,33,34,37], and none assessed multiple exposure durations within the same cohort to determine whether these acute effects scale with cumulative, real-world use. The evidence base for this field is therefore weighted heavily toward acute, single-session experimental designs, and correspondingly light on data addressing chronicity.

Paediatric research in particular remains dominated by cross-sectional and short-term experimental designs, and long-term data on whether early-life digital device exposure predisposes to chronic DED in adulthood are largely absent [37,38].

Potential confounders - including ambient humidity and air conditioning, contact lens wear, pre-existing ocular surface disease, medication use, and, in the paediatric COVID-19 literature specifically, concurrent reductions in outdoor time and altered sleep - are inconsistently measured or adjusted for across studies, making it difficult to isolate the independent contribution of screen exposure itself [11,39,40].

Finally, the published literature may under-represent null or negative findings. Unlike the preceding six points, this is currently asserted rather than demonstrated for this specific literature: no funnel plot or formal test for publication bias in the digital-device-dry-eye literature is available to this review, and it should be treated explicitly as a plausible hypothesis rather than an established finding.

These limitations do not undermine the biological plausibility or overall direction of the association between digital device use and DED, which is well supported mechanistically by direct evidence linking incomplete blinking to both symptomatic and structural disease markers [20,27,28]. They do, however, argue for a specific research agenda. The first priority should be this review’s own argument: characterising the time course over which ocular surface measurements return to baseline after screen exposure ends, and using that to set a standard pre-test interval for dry eye research and clinical assessment. Beyond this, the agenda should include longitudinal cohort studies that track objectively measured, device-logged screen exposure alongside standardised dry eye outcome measures over multiple years; randomised trials of behavioural and pharmacological interventions specifically in high digital device users, adequately powered and blinded where feasible; head-to-head experimental comparisons of different device types and display technologies within the same participants; and dedicated long-term follow-up of children exposed to intensive early-life screen use as they enter adulthood. Until such studies are available, clinicians and researchers alike should treat individual prevalence figures, effect sizes, and management recommendations in this field as provisional rather than definitive.

## Conclusions

Digital device use is one of the most prevalent and consistently identified modifiable risk factors for DED, even though the precise magnitude of its effect remains difficult to establish because of substantial heterogeneity in how both exposure and disease are defined and measured. The central mechanistic link is a rise in incomplete blinking during sustained near-screen tasks, which appears more specifically attributable to the display than the accompanying reduction in blink rate, which instead appears substantially task-driven. Incomplete blinking is associated with more than double the odds of meeting dry eye diagnostic criteria in cross-sectional data, though this data does not itself establish that screen use causes the incomplete blinking observed in habitual users.

This relationship has been demonstrated across computers, smartphones, tablets, and e-readers, with emerging evidence that backlit LCD and OLED technology may carry a greater ocular surface burden than e-paper displays or paper. It has been shown in office workers, in students, and in children, with the strongest recent paediatric evidence showing a clear dose-response relationship with daily device hours and a protective association with time spent reading physical books, and it was thrown into sharp relief by the abrupt increase in screen-based education during the COVID-19 pandemic. No additional effect of contact lens wear on screen-related dryness was detected in a single experimental study using short exposures; this is not evidence that no such effect exists. Artificial tears remain an effective first-line strategy regardless of lens wear status.

Clinicians should incorporate digital device use, including timing relative to any planned ocular surface testing, into the dry eye history. They should counsel patients, particularly children and heavy device users, on refractive correction where indicated and on behavioural strategies including conscious blinking and lubricating drops, while recognising that the 20-20-20 rule has performed poorly when tested directly, that blue-light-blocking lenses lack robust supporting evidence, and that even oral omega-3 supplementation, the only intervention with a positive certainty rating in this field’s strongest management review, is contradicted by a large negative trial in DED more broadly.

The TFOS Lifestyle report has already provided a comprehensive, systematically searched synthesis of this field. What that report identifies, and does not yet answer, is how ocular surface measurements change over the hours after screen use ends, and how much this uncontrolled interval has contributed to the inconsistency running through the literature reviewed above. The objective measurement tools needed to answer this question did not exist when that report’s search closed in 2022. They exist now. Establishing a standard pre-test interval for dry eye research, informed by a properly designed time-course study, is the most direct way this field can now resolve one of its own most persistent sources of measurement error.
